# Effect of Solution pH on the Adsorption of Paracetamol on Chemically Modified Activated Carbons

**DOI:** 10.3390/molecules22071032

**Published:** 2017-06-22

**Authors:** Valentina Bernal, Alessandro Erto, Liliana Giraldo, Juan Carlos Moreno-Piraján

**Affiliations:** 1 Departamento de Química, Universidad Nacional de Colombia, Cra 30 No. 45-03, Bogotá D.C. 110231, Colombia; vbernalf@unal.edu.co (V.B.); lgiraldogu@unal.edu.co (L.G.); 2 Dipartimento di Ingegneria Chimica, dei Materiali e della Produzione Industriale, Università di Napoli Federico II, P. Le Tecchio 80, 80125 Napoli, Italy; aleserto@unina.it; 3 Departamento de Química, Universidad de los Andes, Cra 1a No. 18A-10, Bogotá D.C. 110231, Colombia

**Keywords:** activated carbon, adsorption isotherms, Gibbs energy change, immersion enthalpy, paracetamol

## Abstract

Paracetamol adsorption in acidic, neutral and basic media on three activated carbons with different chemistry surfaces was studied. A granular activated carbon (GAC) was prepared from coconut shell; starting from this sample, an oxidized activated carbon (GACo) was obtained by treating the GAC with a boiling solution of 6 M nitric acid, so to generate a greater number of oxygenated surface groups. In addition, a reduced activated carbon (GACr) was obtained by heating the GAC at 1173 K, to remove the oxygenated surface groups. Paracetamol adsorption was higher for GACr due to the lower presence of oxygenated surface functional groups. Moreover, adsorption was highest at neutral pH. The magnitude of the interactions between paracetamol molecules and activated carbons was studied by measuring the immersion enthalpies of activated carbons in solution of paracetamol at different concentrations and pH values and by calculating the interaction enthalpy. The highest value was obtained for GACr in a paracetamol solution of 1000 mg L^−1^ at pH 7, confirming that paracetamol adsorption is favoured on basic activated carbons at pH values near to neutrality. Finally, the Gibbs energy changes confirmed the latter result, allowing explaining the different magnitudes of the interactions between paracetamol and activated carbons, as a function of solution pH.

## 1. Introduction

Paracetamol, or 4-hydroxyacetanilide, is a widely used drug due to its analgesic and antipyretic properties. It is available by prescription and as an over-the-counter medicine; the U.S. Food and Drug Administration (FDA) calculations indicate that some 24.6 billion doses were sold in 2008 [1].

Easy access to pain medication is considered a public health problem due to the high consumption of drugs, such as paracetamol, by the population. Although this drug is considered safe in the United States, 51% of acute liver failures that occurred between 1998 and 2003 were due to the consumption of paracetamol in high amounts [2,3]. The main physicochemical properties and the structure of paracetamol are presented in Table 1.

The excessive consumption of this drug causes several physiological problems. After administration, distribution, metabolism and excretion, part of the drug that is not metabolised is eliminated through urine. Thus domestic or hospital effluents can contain variable concentrations of this pollutant. The same properties in Table 1 facilitate accumulation of paracetamol in soil and persistence in water [4,5,6].

In wastewater treatment plants, paracetamol is only partially removed from polluted water, as conventional wastewater processing techniques (e.g., biological processes) are not specifically designed for emerging contaminants. Consequently, the presence of bioactive molecules in water for human consumption generates long-term toxicological risks given that the drug can accumulate in adipose tissue to concentrations capable of generating biological activity. For this reason, new research efforts are currently being made to find novel and efficient water treatment methods. Adsorption onto porous solids is a very promising solution to remove organic and inorganic contaminants [7]. Activated carbons are among the most used adsorbents due to their versatility and favourable properties, such as high surface area, porosity and specific chemical properties, which allow interacting with different chemical compounds. Indeed, the efficiency of adsorption on activated carbon strongly depends on the specific interactions between adsorbent and adsorbate, which in turn mainly depend on their chemical properties [7,8,9,10].

This work aims to investigate the adsorption interactions between paracetamol and three activated carbons of different surface chemistry in a wide concentration range (10–1000 mg L^−1^). The solution pH was changed to evaluate the interactions when charges appear in the drug structure (i.e., due to ionisation phenomena) and on the surface of the activated carbon. The immersion enthalpies of the activated carbons in solutions of paracetamol at three different concentrations and three different pH values were determined to further investigate the magnitude of the interactions between the adsorbent and paracetamol. Finally, the process was described from a thermodynamic point of view by the Gibbs energy change of the systems, to give further insights on the adsorption mechanism.

## 2. Results and Discussion

### 2.1. Chemical Characteristics of the Activated Carbons

The physicochemical properties of the prepared activated carbons show that the solids are almost microporous. A slight variation in the textural properties was observed for the different GAC preparation treatments, which is expected to affect the adsorption of paracetamol. Soudani et al. [11] indicate that the decrease of surface area and micropore volume for GACo can be related to the formation of oxygenated groups at the edges of the pore openings, which limits the accessibility of nitrogen molecules into the porous structures, thus decreasing the adsorption. Conversely, the increase in surface area and micropore volume observed for GACr can be ascribed to the lower amount of oxygenated groups on the surface, which were removed during the thermal treatment 1173 K [11]. In order to confirm the effect exerted by the chemical modification on the textural properties and to investigate their influence on the adsorption of paracetamol, Boehm analysis were carried out on all the activated carbon sample (Table 2).

As expected, the GACo has a high quantity of acidic functional groups due to the oxidation and condensation reactions between the functional groups present in the GAC and nitric acid. Compared with GACo, GAC presented a lower quantity of acidic functional groups. The quantity of phenol groups on surface was higher than those present on GACo because the condensation reactions with nitric acid also cause a decrease in the phenol group content. The basicity of the activated carbons can be significantly increased by reducing oxygenated functional groups with temperature due to reach their thermal instability temperature (GACr). Following this path, the different oxygenated groups were transformed into CO_2_ and CO, and their amounts were almost negligible on GACr.

The basicity of the activated carbons is still debated; Boehm’s titration indicates that the basicity of the activated carbon is mainly due to π delocalized electrons on the graphenic layers, forming the activated carbon, and other groups such as pyrones, having low basicity. However, the distribution of the carbonyl group and oxygen that include polycyclic aromatic compounds can increase the number of basic groups [12]. Moreover, GAC and GACo have an acidic pH at the pH_PZC_, lower for GACo, while the GACr sample had a slightly basic value.

### 2.2. Paracetamol Adsorption Test on Activated Carbons

Studying paracetamol adsorption on activated carbon is relevant because this compound is an emerging pollutant which conventional water treatment procedures do not effectively remove. Adsorption is a versatile technique, suitable for further exploration. The adsorption of pharmaceutical compounds from an aqueous phase on activated carbon was mainly due to interactions between the functional groups in the drug structure and the groups on the solid sorbent surface [13]. In this work the effect of pH on the adsorbate-adsorbent interactions and on the distribution of these functional groups was investigated. These characteristics have significant effects on the adsorption capacity.

Weak electrolytes, such as paracetamol, coexist in both ionized (a base) and non-ionised (an acid) forms. The distribution of acid and basic forms is strictly dependent on the solution pH and their interaction with solid may favour or disfavour the adsorption process if forces of attraction or repulsion prevail, respectively.

**Figure 1:**
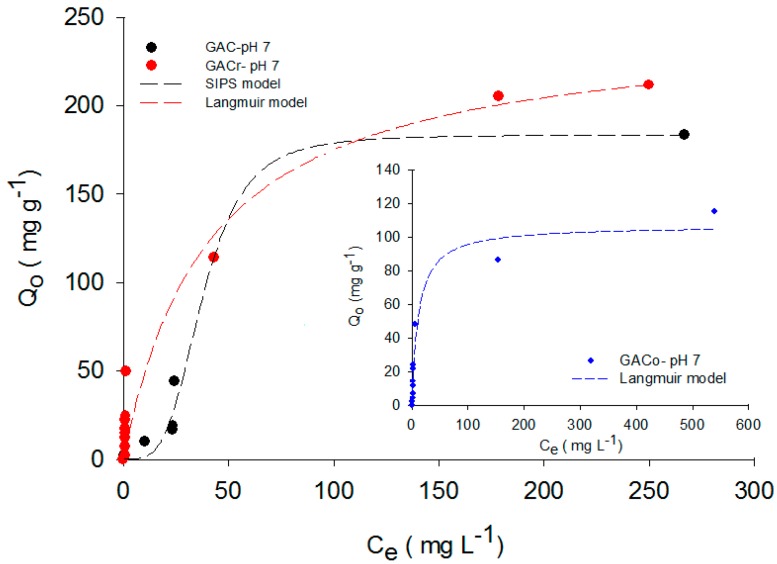
Paracetamol adsorption isotherms on GAC, GACo and GACr at pH 7 and T = 298 K.

The concentration of these conjugates depend on the solution pH and pKa (9.38 for paracetamol, see Table 1) as expressed in the Henderson-Hasselbalch equation. From this equation, the amount of acidic and basic forms can be calculated. For example, 90% of paracetamol is in its protonated form up to pH 7, while in basic medium the phenol group proton is removed, and at pH 11, 90% of the deprotonated base is found. The presence of charges on the activated carbon surface corresponding to a specific solution pH is determined by pH_PZC_. This represents the pH of the solution at which the net surface charge is neutral. Hence, at solution pH below this value, activated carbon surface has an overall positive charge. At solution pH higher than pH_PZC_, the surface of the activated carbon becomes negatively charged, because of the deprotonation of functional groups [14]. Considering all this, it is evident that solution pH exerts a significant influence on paracetamol adsorption on activated carbons. Figure 1 shows the paracetamol adsorption isotherms on the three activated carbons at pH 7 and T = 298 K.

The adsorbed amount of paracetamol is the highest on GACr, followed by GAC and GACo. According to Fuentes et al. [15], this result is due to the highest basicity of GACr associated with increased density of π-delocalized electrons generated during the heat treatment and with basic functional groups that, according to the results of the Boehm titration (Table 2), are the highest for GACr.

Moreover, at pH 7, the pH_PZC_ of GACr is higher (cf. Table 2), the surface is positively charged and the paracetamol is mainly in the neutral form, hence neither repulsion nor adsorption is favoured [9].

Terzyk [16] affirms that the adsorption of aromatic compounds from aqueous solution on activated carbon can occur according to three different mechanisms: dispersive interactions by π electrons, hydrogen bond formation and donor-acceptor electron complexes. Paracetamol adsorption on granular activated carbon is expected to involve all these mechanisms. However, the adsorption due to π electron interactions seems to be predominant because the oxygen functional groups capable of forming Lewis acid-base complexes or hydrogen bonds are lower on activated carbons with modified surface chemistry [16].

**Figure 2:**
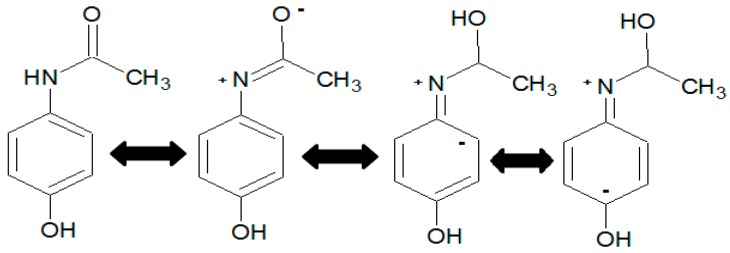
Resonance structures in a paracetamol molecule.

On the contrary, GACo shows a lower adsorption capacity because it has a high concentration of acidic groups, the surface polarity is increased and hydrogen bond can be formed with water molecules, which have a greater proportion and higher polarity than paracetamol [17]. The paracetamol molecule shows resonance structures of the free electron pair of the nitrogen atom, as shown in Figure 2.

The formation of an electron acceptor-donor complex is possible between the groups with free electron pairs, such as oxygen of the phenol groups of activated carbons, and the electropositive nitrogen in paracetamol molecule, indicating that the decrease in the adsorption capacity can be related to the decline of these groups in the oxidized activated carbon. The paracetamol molecule has two proton acceptor and donor groups. However, due to the changes generated by the resonance, the nitrogen passes from proton acceptor to donor. Therefore, hydrogen bond interactions will be higher in activated carbon with higher concentrations of proton acceptors groups (i.e., GACr) [16].

The π electron interactions are relevant for the adsorption of aromatic compounds. However, the influence exerted by the substituent type present in the aromatic molecule, such as paracetamol, should also be considered. In fact, the amide group present in paracetamol is an activating group of the aromatic ring, whereby π electrons in the molecule are available to generate interactions with the delocalised electrons of activated carbon.

**Figure 3:**
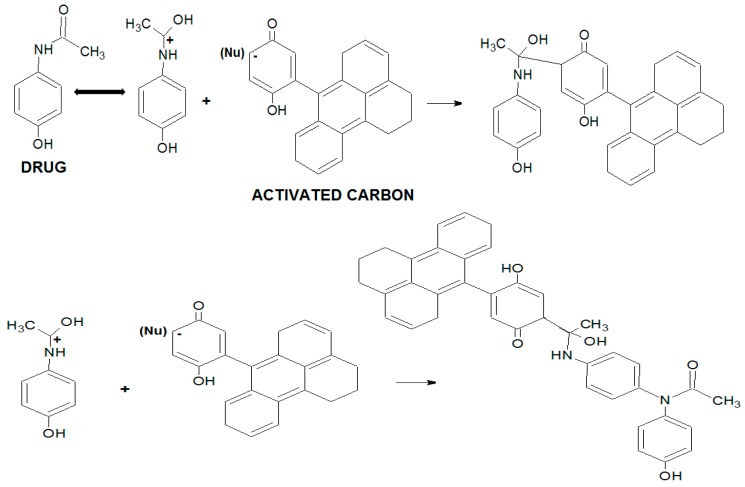
Nucleophilic addition reaction between activated carbon and paracetamol.

As previously stated, the adsorption process of pharmaceutical compounds, such as paracetamol, is strictly influenced by electrostatic interactions between adsorbent and adsorbate. Therefore pH changes in the medium may generate changes in the adsorbate structure and activated carbon physicochemical properties. In acidic medium, the paracetamol molecule presents a nitrogen resonance electron pair, as shown in Figure 2. The carbocation generation in the sp^2^ carbon of the amide group facilitates the interaction with the proton acceptor groups of activated carbon, as well as nucleophilic addition with surface groups, such as hydrogen sulfides, trisubstituted amines and hydroxyls (Figure 3) [8,18].

**Figure 4:**
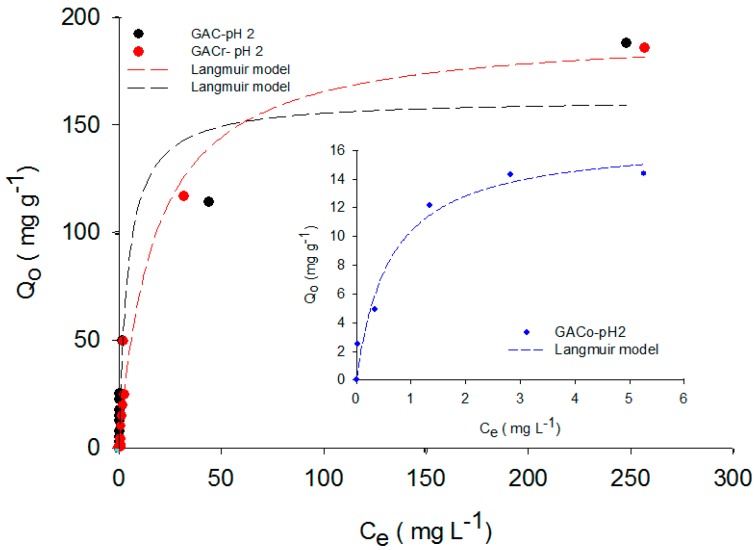
Paracetamol adsorption isotherm on GAC, GACo and GACr at pH 2 and T = 298 K.

The nucleophilic addition reactions occur not only with activated carbon groups, in acidic medium. Paracetamol dimerization can also occur due to nucleophilic addition to the phenolic groups present in molecules of the same species [19]. As the solution pH can exert a significant influence on paracetamol adsorption, a dedicated study was carried out. Figure 4 shows the paracetamol adsorption isotherms in acidic medium (pH 2) onto GAC, GACo and GACr.

**Figure 5:**
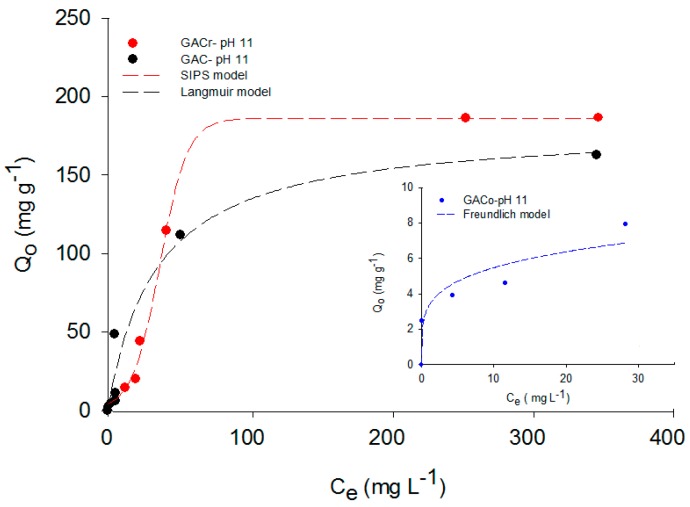
Paracetamol adsorption isotherms on GAC, GACo and GACr at pH 11 and T = 298 K.

Compared with the data at pH 7, a decrease in the adsorption capacity at lower pH for all the investigated adsorbents was observed, possibly due to the formation of paracetamol dimers in the solution and to the repulsions between the activated carbon surface having positive charges as well as the carbocation of paracetamol. GACo had the lowest performance, even when lower concentrations were used. In all cases, the activated carbon surface was polarized due to the hydronium ions present, favoring hydrogen bond formation with the solvent [17,19]. As adsorption capacity decreases with decreasing solution pH, it can be confirmed that the donor-acceptor electron mechanism is less active in this adsorption system. In order to complete the analysis of pH effect, in Figure 5, paracetamol adsorption isotherms at pH 11 are reported.

The ranking between adsorbents was maintained in this instance, and GACr showed the highest adsorption capacity. However, GACo and CACr showed a significant reduction in paracetamol adsorption capacity, when compared to data at neutral pH (Figure 1). Indeed, at pH 11, paracetamol was present in its dissociated anionic form and the surface of all the activated carbons were negatively charged. Hence, repulsion phenomena are predominant and determine the observed reduction in adsorption capacity. On contrast, GAC had similar adsorption capacity at pH 11 compared to adsorption at neutral pH. However, the processes are described by different models, this may be due to a slight curvature at low concentrations of paracetamol at pH 11 due to the heterogeneity of the adsorbents accentuated by the presence of hydroxyl groups in the medium, which interact with the surface acid groups of the adsorbate. Differently, at paracetamol concentrations higher than 200 mg L^−1^ the isotherms could be described with the same mathematical model.” Thus, paracetamol adsorption on GAC seems to be less dependent on specific interactions between functional groups on the activated carbon surface and the adsorbate.

The effect of pH on paracetamol adsorption has not been widely studied. Ferreira et al. [18] studied paracetamol adsorption at pH values of 2, 6.5 and 11 on activated carbon prepared from coconut shell, whose properties are comparable to those of the GAC investigated in the present work. Similar to the result obtained in the present study, they determined that adsorption is highest at pH 6.5, at which a 75 mg g^−1^ adsorption capacity was achieved in paracetamol solutions with an initial concentration of 50 mg L^−1^, while at pH 11, the adsorption capacity decreased to 43 mg g^−1^.

The results obtained in the present study were consistent with those reported Galhetas et al. [19], which confirmed that paracetamol adsorption was favoured for reduced activated carbons and in neutral pH solutions.

In order to extend the analysis of the entire adsorption data set, paracetamol adsorption isotherms were adjusted to the Langmuir, Sips and Freundlich models. The modelling results are reported in Figure 1, Figure 4 and Figure 5 for adsorption data at neutral, acid and basic pH values, respectively. For each adsorption isotherm, the best fitting model was reported.

The Langmuir model assumes that the maximum adsorption capacity corresponds to a monolayer of adsorbate molecule on the adsorbent surface. It is also assumed that adsorbate molecules bind to specific sites and each site accommodates one molecule. It is further assumed that the adsorptive energy is equal for all sites regardless of adsorbed molecules in neighboring sites, the adsorbent surface is flat, smooth and adsorbate-adsorbate interactions are negligible. Equation (1) describes the Langmuir model [20,21]:

(1) $$q_{e}=\frac{Q_{m}K_{L}C_{e}}{1+K_{L}C_{e}}$$

where *q_e_* represent the paracetamol adsorption quantity, *Q_m_* is the maximum adsorption capacity and corresponds to the monolayer, *C_e_* is the paracetamol concentration in equilibrium and *K_L_* is the Langmuir constant.

The Freundlich model is an empirical model, and it is frequently used to describe the adsorption of organic compounds in aqueous solution. It hypothesizes an exponential decay in the distribution of the adsorption energies of the active sites. Its mathematical representation is given in Equation (2) [20,21]:

(2) $$q_{e}=K_{F}C_{e}^{1/n}$$

where the constants *K_F_* and *n* depend on the interaction adsorbent-solute and on the temperature. The *n*^−1^ values may be less or greater than unity, when the value is less than unity indicate a favourable adsorption.

The Sips model combines the Langmuir and Freundlich expressions to predict the adsorption in heterogeneous systems. Equation (3) shows the mathematical expression of this model [21]:

(3) $$q_{e}=\frac{Q_{m}K_{s}C_{e}^{n}}{1+K_{s}C_{e}^{n}}$$

where *Q_m_* is the maximum adsorption capacity, *K_s_* and *n_s_* are constants.

At low concentrations, Equation (3) is reduced to the Freundlich model, while at high concentrations; the maximum adsorption capacity corresponds to monolayer formation, as indicated by Langmuir model. The model parameters are directly related to the variations in the system properties, such as pH, concentration and temperature. Table 3 shows the best fitting models and the relative parameters determined for paracetamol adsorption on the three activated carbons at different pH values.

The maximum adsorption capacity was determined by adjusting the data of the mentioned models and, as expected, paracetamol adsorption at pH 7 was the highest for all the activated carbons. For example, for GACo and GAC, the paracetamol adsorbed quantity at pH 2 and 11 was reduced to the tenth of the adsorbed amount at pH 7.

From a thermodynamic point of view, the adsorption process involves the loss of degrees of freedom due to the transition of adsorbate from three to two dimensional phase. This transition or loss in degrees of freedom quantitatively determined the values of the thermodynamic potentials, Gibbs energy, entropy and enthalpy.

In a heterogeneous system such as activated carbon, Gibbs energy decreases due to the reduction in the attractive force balance on the adsorbent surface. Thus, the process is spontaneous. By reducing the adsorbate’s degrees of freedom, the entropy change is negative. Therefore, from Equation (4) it is clear that the adsorption enthalpy change is negative [22]:

(4) ΔG=ΔH−TΔS

The thermal effects of paracetamol adsorption on activated carbon can be determined by immersion calorimetry. This technique measures the energy changes associated with water molecule desorption from the activated carbon surface and subsequent paracetamol adsorption after contact with the activated carbon [23].

Immersion enthalpy is produced by specific and non-specific active interactions during adsorption and is considered an important parameter to fully characterize solid-liquid systems. Its value depends on multiple factors such as surface area and adsorbent polarity [24]. Equation (5) describes the immersion enthalpy in terms of the interactions occurring in the system.

(5) $$\Delta H_{imm}=\Delta H_{ads-GAC}+\Delta H_{solv-GAC}+\Delta H_{sol-solv}$$

where $\Delta H_{ads-GAC}$ represents the interactions asdorbate-activated carbon, $\Delta H_{solv-GAC}$ the interactions solvent-activated carbon and $\Delta H_{solv-solv}$ the interactions solvent-solvent. The interactions adsorbate-adsorbate can be neglected with good approximation.

The interaction enthalpy corresponding to the energy produced by the contact between the adsorbate and the adsorbent, also neglecting the solvent-solvent interactions, is determined by Hess’s Law from the immersion enthalpy and activated carbon-solvent interactions:

(6) $$\Delta H_{int}=\Delta H_{ads-GAC}=\Delta H_{imm}-\Delta H_{solv-GAC}$$

The two values of immersion enthalpy show the differences in the interactions between the adsorbate and the activated carbon with different functional groups on the surface and, in association with Gibbs energy and entropy values, can supplement the information provided by adsorption isotherms [24]. Table 4 shows the paracetamol immersion and interaction enthalpies on the three activated carbons at six different paracetamol concentrations (10, 50, 100, 200, 500 and 1000 mg L^−1^).

In general, the immersion enthalpy increases (in absolute value) with this ranking GACo > GAC > GACr, confirming the influence of the carbon surface functionalities in the network of phenomena occurring in solution. The magnitude of interaction enthalpy follows an opposite trend, which can be ascribed to the different interactions of the solvent (water) with the activated carbons. The interaction once again confirmed the influence of surface functional groups. In fact, the ranking of water immersion enthalpy $\left(\Delta H_{solv-GAC}\right)$ at neutral pH was GACo > GAC > GACr, and the values were −66.6, −49.7 and −32.4 J g^−1^. This result is due to the increased interaction with the solvent and the hydrogen bond formation between water and oxygen groups on the adsorbent surface. In basic medium, the enthalpy values follow the same trend as at neutral pH (−97.5, −57.3 and −35.1 J g^−1^ for GACo, GAC and GACr, respectively). These values reflect the acid-basic interactions that occur between the acidic functional groups on the activated carbons and the hydroxyls in the medium. Moreover, GACr has a notably lower value due to the absence of carboxylic functional groups. In acidic medium, the behavior of immersion enthalpy is opposite at pH 11. The GACr has an enthalpy value of −58.2 J g^−1^, followed by GAC (−51.4 J g^−1^), and finally GACo (−42.8 J g^−1^).

As an example, GACr in a paracetamol solution of 1000 mg L^−1^ at pH 7 presented an immersion enthalpy value of −36.0 J g^−1^. For the same system, the interaction enthalpy was 68.4 J g^−1^, confirming that paracetamol adsorption is favoured on basic activated carbons at neutral pH values, due to multiple factors, such as a: (a) decrease in the competitive adsorption with the solvent due to less hydrogen bond formation; (b) lower excess of protons that interact with π electrons on the graphenic layers and the aromatic ring of paracetamol, facilitating the donor-electron acceptor complex formation. The interaction enthalpies were positive, possibly because paracetamol adsorption is associated with the desorption of water or solvent from the activated carbon surfaces and this requires energy from the surroundings.

**Figure 6:**
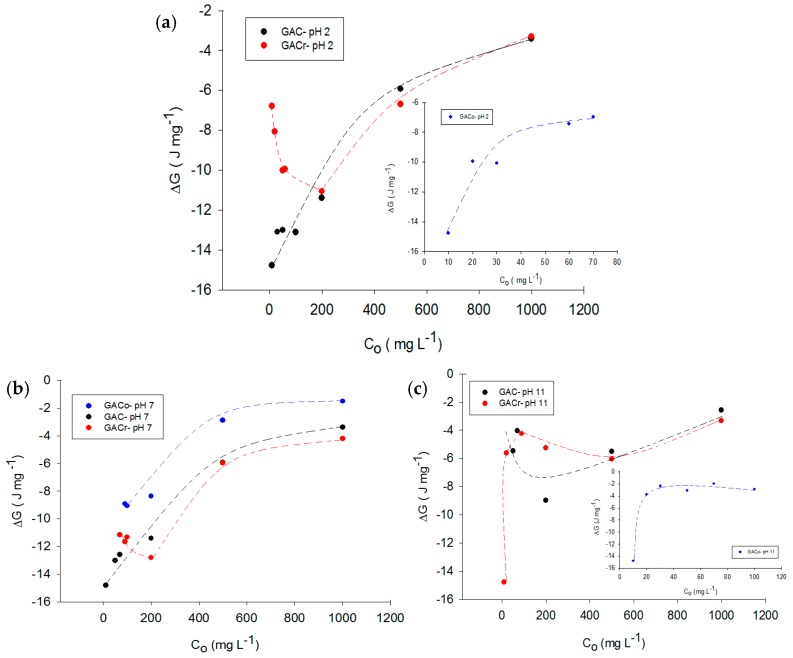
Gibbs energy change for paracetamol adsorption on activated carbons at (**a**) pH 2, (**b**) pH 7 and (**c**) pH 12. Dotted lines represent the process tendency.

In order to give a deeper insight on adsorption mechanism, Gibbs energy change of the systems were also analyzed. Gibbs energy change of paracetamol adsorption on activated carbon provides information about the spontaneity of the process, as well as changes in the chemical potential of the system when there are variations in conditions, such as concentration and pH. The Dubinin-Radushkevich equation expresses the chemical potential for adsorption in the aqueous phase as [25]:

(7) $$\Delta \mu =-RTLn\frac{C_{o}}{C_{e}}$$

where *C_o_*/*C_e_* represents the ratio between the initial and equilibrium concentration, *R* is the universal gas constant and *T* is temperature in Kelvin. The chemical potential corresponds to molar Gibbs free energy of a component. Figure 6a–c depict the Gibbs energy change for paracetamol adsorption on the three types of activated carbon at acidic, neutral and basic medium, respectively.

As expected, all Gibbs free energy changes were negative, as adsorption is spontaneous phenomenon. Figure 6 shows the change in Gibbs energy. For GACr, adsorption is favoured at neutral pH, as under these conditions adsorbate-adsorbent interactions increase the enthalpic component, which is directly related to Gibbs energy change (see Equation (7)).

The potential for GACr, at pH 2 and 7 decreases at low paracetamol concentrations and then increases with increased paracetamol concentration. This trend is due to the presence of few adsorbate molecules in solution at low concentrations, which leads to the desorption of solvent molecules from the surface of the adsorbent. Paracetamol adsorption requires less energy from the surroundings. When the paracetamol concentration increases the energy also increases changes due to the formation of adsorbate-adsorbent interactions. At pH 11, Gibbs energy change increases almost monotonically in all systems. For GACo, Gibbs energy change are qualitatively independent of the pH, as an increase in the equilibrium concentration determines a decrease in Gibbs energy, due to an increase in interactions between functional groups on the activated carbon and the adsorbate. A decrease in pH is related to an increase in the Gibbs energy change because at acid pH a greater amount of hydronium ions in the medium are adsorbed by activated carbon, decreasing the adsorption and interaction capacity of paracetamol.

Finally, for GAC, no single trend for change of Gibbs energy with pH was found, due to the amphoteric characteristics of the surface. At pH extreme values of 2 and 11, the change in Gibbs energy was values above the calculated energy change for the process at pH 7 under the same equilibrium concentration. This indicates that pH change did not favour the adsorption process in this activated carbon.

## 3. Materials and Methods

### 3.1. Activated Carbons

A commercial Carbochem brand GS50 activated carbon (CARBOCHEM INC., Philadelphia, PA, USA) was preliminary treated by immersion in concentrated hydrochloric acid, washed with distilled water until a constant pH and dried at 100 °C. This sample is referred as GAC. Starting from this sample, an oxidized activated carbon (GACo) was produced by treatment with a nitric acid solution 6 M for 6 h at its boiling temperature. Similarly, a reduced activated carbon (GACr) was obtained by heating the raw GAC for 2 h at 1173 K under N_2_ flux. All the activated carbons were fully characterised by N_2_ adsorption at −77 K. For GAC, the surface area was 842 m^2^ g^−1^ and the micropore volume was 0.35 cm^3^ g^−1^. For GACo, the surface area was 816 m^2^ g^−1^ and the micropore volume was 0.32 cm^3^ g^−1^. Finally, the GACr showed a surface area equal to 876 m^2^ g^−1^ and the micropore volume was 0.34 cm^3^ g^−1^.

### 3.2. Chemical Characterization

The acidity and basicity for different types of activated carbons were evaluated by the well-known Boehm titration method, consisting of a back-titration of the functional groups on the activated carbon [12]. The Boehm methodology is based on quantifying different groups from titrations with bases of different strength. In particular, NaOH solutions were used to titrate the sum of carboxylic acids, phenols and lactones. Na_2_CO_3_ was used to titrate carboxylic acids and lactones. NaHCO_3_ was used to titrate carboxylic acids. The difference in titration volumes allowed the quantification of the single functional groups. Finally, the basic groups were quantified by titration with HCl as a total sum.

In details, 500 mg of each activated carbon were weighed and added to 50 mL of either 0.1 M NaOH, Na_2_CO_3_ or NaHCO_3_ solutions. Similarly, the addition of 50 mL of 0.1 M HCl solution to new samples of activated carbons allowed determining the total basicity. The mixtures were stored at 298 K under constant agitation for 5 days. After this, 10 mL aliquots of each solution supernatant were sampled and titrated with NaOH or HCl solution, for the quantification of basic and acid functional groups, respectively [12]. The pH at point of zero charge (pH_PZC_) was determined by mass titration. Specifically, 50–500 mg of activated carbon were weighed and placed in receptacles with aliquots of 10 mL of 0.1 M NaCl added. The samples were stored under agitation (150 rpm) and constant temperature (20 °C ± 2 °C) for 48 h. Finally, the pH of each solution was measured [26].

### 3.3. Adsorption Tests

Paracetamol solutions were prepared with reagent grade paracetamol of 98% purity (ALPHA, Great Britain) and distilled water, in concentrations ranging between 10 and 1000 mg L^−1^. For acid solution preparation, a defined amount of 0.01 M HCl solution was added. Similarly, different amounts of 0.001 M NaOH solution were used to prepare basic paracetamol solutions.

For the determination of paracetamol adsorption isotherms, 100 mg of each activated carbon was weighed in amber glass containers and 25 mL of paracetamol solution was added. The containers were kept at constant temperature (298 K) under stirring until equilibrium was reached. Then, the solutions were filtered and the equilibrium concentration determined by UV-vis spectrophotometry on a GENESYS 10 UV-vis scanning apparatus (Thermo Fisher Scientific, Madison, WI, USA) at a maximum wavelength of either 242 nm (for acidic and neutral pH tests) or 256 nm (basic pH tests). The experimental data were modeled with the statistical program SigmaPlot 10^®^ (Systat Software Inc., San Jose, CA, USA)

### 3.4. Determination of Immersion Enthalpies

Immersion enthalpies of activated carbon in paracetamol solutions, water, hydrochloric acid and sodium hydroxide solutions were carried out in a Tyan-Calvet type heat conduction microcalorimeter, which was equipped with a stainless steel cell of 15 mL capacity, in which 10 mL of the immersion liquid was placed. A quantity of 100 mg of each activated carbon was weighed in a glass ampoule with a fragile tip and placed in the calorimetric cell. The electric potential was recorded and the increasing of value due to wetting of the solid was recorded.

## 4. Conclusions

Adsorption of paracetamol, an analgesic considered as an emergent contaminant, on different activated carbons with modified chemical surfaces was studied. Its adsorption decreased when the oxidation degree of the activated carbon increased due to the competitive effect of water. The adsorption capacities at pH 7 were highest on the three types of activated carbon. At acidic pH, the activated carbons were positively charged and repulsion with the carbocation of paracetamol or the dimers formed could occur. At basic pH, the decrease in adsorption capacity was directly related to the repulsion between the negatively charged activated carbon surface and the anionic (deprotonated) paracetamol. For all the adsorption isotherms, a modelling analysis was carried out by the Freundlich, Langmuir and Sips models and the best fitting models, together with their parameters determined.

The interactions between paracetamol molecules and activated carbon were further studied by immersion calorimetry. In general, the immersion enthalpy increased (in absolute value) with this ranking GACo > GAC > GACr, confirming the influence of the carbon surface functionalities in the network of phenomena occurring in solution. The values of enthalpy of interaction follow an opposite trend, which can be ascribed to the different interactions of the solvent (water) with the activated carbons, once again confirming the influence of surface functional groups. The immersion enthalpy corresponded to the highest adsorption capability were −36.0 and −68.4 J g^−1^, due to the adsorption of paracetamol solution of 1000 mg L^−1^ at pH 7on GACr. The lowest value of immersion enthalpy (34.8 and −37.4 J g^−1^) was recorded for GACo with paracetamol solutions of 10 mg L^−1^ at pH 10 and 2, respectively

State function Gibbs energy was determined for paracetamol adsorption at pH 2, 7 and 11, and for the three types of activated carbons. In all cases, Gibbs energy changes were negative, indicating that the process was spontaneous. For GACo, an increase in paracetamol concentration was associated with a decrease in Gibbs energy, which for all systems was between 2 and 10 J mg^−1^. For GAC and GACr, Gibbs energy change depend on the pH value. At pH 11 the interactions adsorbate-adsorbent decrease and it was confirmed that, for GACr, adsorption is favoured at neutral pH.

Finally, paracetamol adsorption on activated carbon was associated with the formation of acid-base, ion-dipole and electron donor-acceptor complex interactions. Each mechanism is relevant at particular pH values. The π-π interactions between reduced activated carbon and Paracetamol are present at all pH values due to the aromatic rings in activated carbon and adsorbent.

## Figures and Tables

**Table 1:** Physicochemical properties of paracetamol.

Drug	Structure *	Molecular Weight (g mol ^−1^)	LogP	So H_2_O (25 °C mg mL^−1^)	pKa
Paracetamol	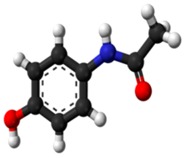	151.2	0.46	14	9.38

* The black spheres correspond to carbon atoms, the red spheres to oxygen atoms, the blue spheres to nitrogen atoms and the white spheres to hydrogen atoms.

**Table 2:** Surface chemical groups on GAC, GACo and GACr activated carbons.

Activated Carbon	Carboxylic Groups (Molecules nm^2^)^−1^	Lactonic Groups (Molecules nm^2^)^−1^	Phenolic Groups (Molecules nm^2^)^−1^	Total Acidity (Molecules nm^2^)^−1^	Total Basicity (Molecules nm^2^)^−1^	pH_PZC_
GACo	0.197	0.039	0.054	0.290	0.036	3.4
GAC	0.029	0.029	0.061	0.141	0.065	5.4
GACr	0.00	0.008	0.029	0.032	0.141	8.9

**Table 3:** Best model fitting and model parameters for paracetamol adsorption on GAC, GACo and GACr activated carbons at different pH values.

pH Value		Best Fitting Model	*Q_m_* (mg g^−1^)	*n*	K	*R*^2^
pH 2	GACo	Langmuir	16.8	N/A	1.6144 L mg^−1^	0.99
	GAC	Langmuir	162.0	N/A	0.2350 L mg^−1^	0.97
	GACr	Langmuir	193.8	N/A	0.0583 L mg^−1^	0.99
pH 7	GACo	Langmuir	106.9	N/A	0.0856 L mg^−1^	0.98
	GAC	SIPS	183.4	0.2648	0.0265 (L mg^−1^) ^1/n *^	0.99
	GACr	Langmuir	245.7	N/A	0.0247 L mg^−1^	0.98
pH 11	GACo	Freundlich	9.06	4.59	3.321( mg g^−1^)	0.94
	GAC	Langmuir	180.6	N/A	0.0288 L mg^−1^	0.98
	GACr	SIPS	186.2	0.1017	0.0269 (L mg^−1^) ^1/n *^	0.99

N/A = Not applicable. * n is the isotherm parameter.

**Table 4:** Immersion and interaction enthalpies of paracetamol on activated carbons at different solution pH.

Paracetamol Concentration (mg L^−1^)	Sample	pH 2		pH 7		pH 11	
		$*\;\Delta H_{imm}$ (J g^−1^)	$\Delta H_{int}\;$ (J g^−1^)	$*\;\Delta H_{imm}$ (J g^−1^)	$\Delta H_{int}$ (J g^−1^)	$*\;\Delta H_{imm}$ (J g^−1^)	$\Delta H_{int}$ (J g^−1^)
10	GACr	−21.8	36.4	−10.3	22.1	−15.8	15.6
	GAC	−16.6	34.8	−8.82	40.9	−14.2	43.1
	GACo	−37.4	5.44	−34.7	31.9	−32.7	64.8
50	GACr	−25.9	32.6	−8.29	24.1	−11.9	19.5
	GAC	−21.7	29.7	−11.6	38.1	−16.5	40.8
	GACo	−43.8	−1.03	−30.7	35.9	−31.8	65.7
100	GACr	−19.9	38.3	−8.89	23.5	−9.21	22.2
	GAC	−20.6	30.8	−8.61	41.1	−27.4	29.9
	GACo	−30.7	12.1	−29.1	37.5	−25.2	72.3
200	GACr	−41.8	16.4	−33.1	−0.73	−10..3	21.1
	GAC	−48.5	2.93	−9.71	39.9	−25.8	31.5
	GACo	-	-	−45.5	21.1	-	-
500	GACr	−47.2	11.0	−34.6	−2.23	−50.6	−19.1
	GAC	−61.1	−9.68	−31.7	18.0	−26.4	30.9
	GACo	-	-	−55.5	11.1	-	-
1000	GACr	−50.4	7.77	−36.0	−3.62	−43.1	−11.6
	GAC	−74.4	−22.9	−54.3	−4.62	−38.0	19.3
	GACo	-	-	−63.7	2.90	-	-

* Immersion enthalpies have standard deviations between 0.11 and 1.79 J g^−1^.
